# Cystine-dependent antiporters buffer against excess intracellular reactive sulfur species-induced stress

**DOI:** 10.1016/j.redox.2022.102514

**Published:** 2022-10-17

**Authors:** Masahiro Akiyama, Takamitsu Unoki, Hanako Aoki, Akiyuki Nishimura, Yasuhiro Shinkai, Eiji Warabi, Kazuhiro Nishiyama, Yuka Furumoto, Naohiko Anzai, Takaaki Akaike, Motohiro Nishida, Yoshito Kumagai

**Affiliations:** a Faculty of Medicine, University of Tsukuba, Ibaraki, 305-8575, Japan; b Faculty of Pharmacy and Graduate School of Pharmaceutical Science, Keio University, Tokyo, 105-8512, Japan; c Department of Basic Medical Sciences, National Institute for Minamata Disease, Kumamoto, 867-0008, Japan; d Graduate School of Comprehensive Human Sciences, University of Tsukuba, Ibaraki, 305-8575, Japan; e National Institute for Physiological Sciences, National Institutes of Natural Sciences, Aichi, 444-8787, Japan; f Graduate School of Pharmaceutical Sciences, Kyushu University, Fukuoka, 812-8582, Japan; g Graduate School of Medicine, Chiba University, Chiba, 260-8670, Japan; h Graduate School of Medicine, Tohoku University, Miyagi, 980-8575, Japan

**Keywords:** Sulfur stress, Cystine, Cysteine persulfide, Antiporter, Cystathionine gamma-lyase

## Abstract

Reactive sulfur species (RSS) play a role in redox homeostasis; however, adaptive cell responses to excessive intracellular RSS are not well understood. Therefore, in this study, we generated transgenic (Tg) mice overexpressing cystathionine gamma-lyase (CSE) to produce excessive RSS. Contrary to expectations, tissue concentrations of RSS, such as cysteine persulfide (CysSSH), were comparable in both wild-type and CSE Tg mice, but the plasma concentrations of CysSSH were significantly higher in CSE Tg mice than in wild-type mice. This export of surplus intracellular RSS was also observed in primary hepatocytes of CSE Tg mice. Exposure of primary hepatocytes to the RSS generator sodium tetrasulfide (Na_2_S_4_) resulted in an initial increase in the intracellular concentration of RSS, which later returned to basal levels after export into the extracellular space. Interestingly, among all amino acids, cystine (CysSSCys) was found to be essential for CysSSH export from primary mouse hepatocytes, HepG2 cells, and HEK293 cells during Na_2_S_4_ exposure, suggesting that the cystine/glutamate transporter (SLC7A11) contributes, at least partially, to CysSSH export. We established HepG2 cell lines with knockout and overexpression of SLC7A11 and used them to confirm SLC7A11 as the predominant antiporter of CysSSCys and CysSSH. We observed that the poor efflux of excess CysSSH from the cell enhanced cellular stresses induced by Na_2_S_4_ exposure, such as polysulfidation of intracellular proteins, mitochondrial damage, and cytotoxicity. These results suggest the presence of a cellular response to excess intracellular RSS that involves the extracellular efflux of excess CysSSH by a cystine-dependent transporter to maintain intracellular redox homeostasis.

## Abbreviations

CSE: cystathionine gamma-lyase
CysSH: cysteine
CysSSCys: cystine
CysSSH: cysteine persulfide
CysSSSH: cysteine polysulfide
Nrf2: nuclear factor-erythroid factor 2-related factor 2
GSH: glutathione
GSSH: glutathione persulfide
SLC: solute carrier
Tg: transgenic

## 1 Introduction

Cysteine (CysSH) is a sulfur-containing amino acid and plays a role in redox homeostasis. While CysSH readily undergoes oxidation to cystine (CysSSCys) in the extracellular space, CysSSCys is imported into the cell by the cystine/glutamate transporter, also known as solute carrier (SLC) family 7 member 11 (SLC7A11), which couples the process to the export of intracellular glutamic acid [1]. Intracellular CysSSCys is then reduced to CysSH, which is a substrate for synthesis of the antioxidant glutathione (GSH) and contributes to cellular redox signaling [1]. Current consensus is that reactive sulfur species (RSS), such as CysSH persulfide (CysSSH), GSH persulfide (GSSH), and their polysulfides, exhibit high antioxidant and nucleophilic activities because the p*K*_a_ values of CysSSH and GSSH are relatively low compared with those of CysSH and GSH [2]. For this reason, RSS are potential repressors of oxidative and electrophilic stresses [[3], [4], [5]]. For example, GSSH has higher hydrogen peroxide scavenging activity than its parent compound, GSH [6]. Pre-treatment with alkylsulfenyl thiocarbonates, which are hydropersulfide precursors, protects against hydrogen peroxide-mediated toxicity in H9c2 cardiac myoblasts [7].

We previously reported that treatment with sodium tetrasulfide (Na_2_S_4_), which is a polysulfide containing sulfane sulfur atoms, diminishes xenobiotic electrophile-mediated covalent modification of cellular proteins, redox signaling pathways, and cytotoxicity through formation of sulfur adducts [[8], [9], [10]]. However, cellular adaptive response systems against intracellular excessive RSS are not well understood. In the present study, excess RSS was modeled in two ways: 1) using transgenic (Tg) mice overexpressing cystathionine gamma-lyase (CSE), an RSS-producing enzymes [6,11]; and 2) by treating various cell cultures with Na_2_S_4_, an RSS donor.

## 2 Material and methods

### 2.1 Materials

MEM essential amino acids solution, MEM non-essential amino acids solution, l-cystine, l-arginine, l-histidine, l-Isoleucine, l-leucine, l-lysine, l-methionine, l-phenylalanine, l-threonine, l-tryptophan, l-tyrosine, l-valine, William's Medium E (WME), Dulbecco's modified Eagle's medium (DMEM), and hanks' balanced salt solution (HBSS), were purchased from FUJIFILM Wako Pure Chemical Corporation (Tokyo, Japan). Proteinase inhibitor cocktail was purchased from Nacalai Tesque, (Kyoto, Japan). Na_2_S_4_ was purchased from Dojindo Molecular Technologies, Inc (Kumamoto, Japan). β-(4-hydroxyphenyl)ethyl iodoacetamide (HPE-IAM) was purchased from Molecular Biosciences (CO, USA). All other reagents and chemicals were of the highest grades available.

### 2.2 Animals and treatment

**Fig. 1:**
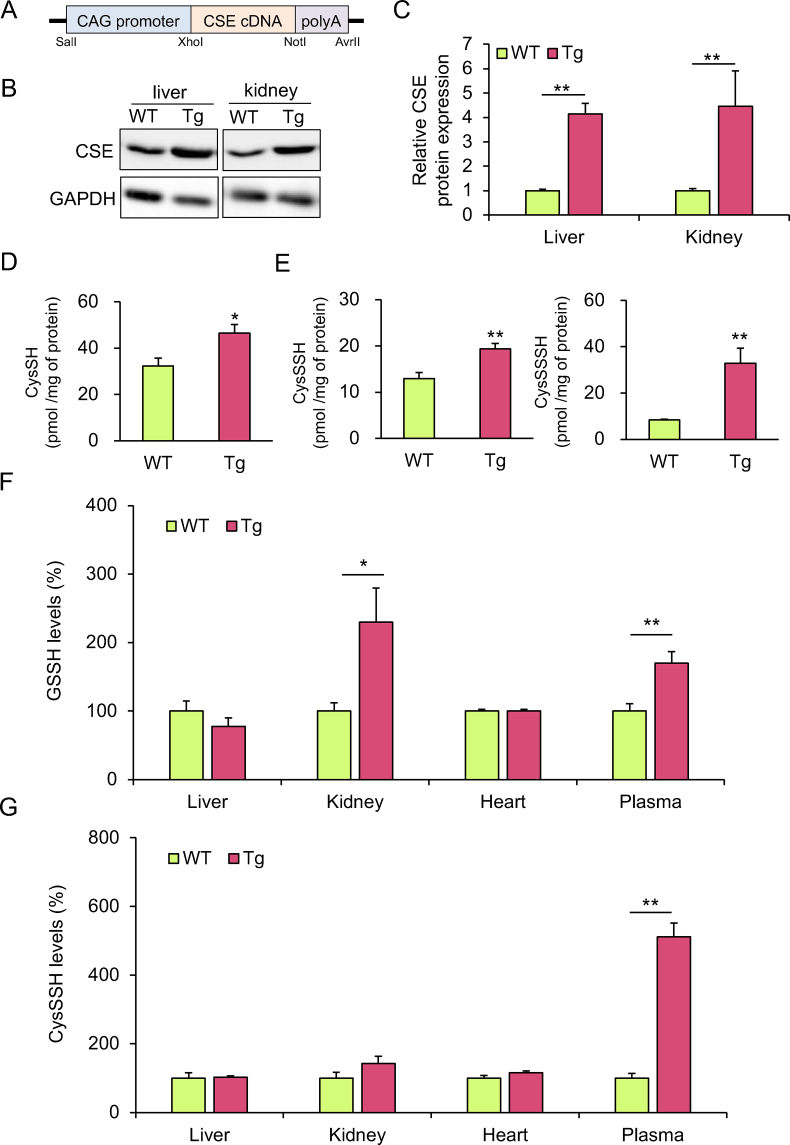
Intra- and extra-cellular concentrations of persulfides in WT and CSE Tg mice. (A) Schematic drawing of the vector used to generate transgenic (Tg) mice overexpressing CSE (CSE Tg mice). CSE protein expression levels in the liver and kidney of wild-type (WT) or CSE Tg mice were (B) analyzed using western blotting (representative data from three determinations are shown) and (C) quantified using densitometry. The ability of the high-molecular-weight protein fraction from the liver of WT and Tg mice to produce (D) cysteine (CysSH) from 1 mM cystathionine or (E) cysteine persulfide (CysSSH) and cysteine polysulfide (CysSSSH) from 1 mM CysSSCys was quantified using LC-MS/MS. *p < 0.05, **p < 0.01. LC-ESI-MS/MS was used to quantitate percentage changes in (F) glutathione persulfide (GSSH) and (G) CysSSH concentrations in the low-molecular-weight fractions of liver, kidney, and heart lysates, and in plasma. Data represent the mean ± SEM (*n* = 3) and are expressed relative to the baseline measured in WT mice. *p < 0.05, **p < 0.01. Sulfur nucleophile concentrations (pmol/mg) in liver tissue from WT mice: CysSSH (0.18 ± 0.028) and GSSH (1.69 ± 0.2); kidney tissue from WT mice: CysSSH (13.7 ± 2.2) and GSSH (0.13 ± 0.01); heart tissue from WT mice: CysSSH (0.69 ± 0.06) and GSSH (1.21 ± 0.03). Sulfur nucleophile concentrations (nM) in plasma from WT mice: CysSSH (284 ± 40) and GSSH (50.2 ± 5.2).

Wild-type (WT) C57BL/6J mice were purchased from CLEA Japan, Inc. (Tokyo, Japan). CSE Tg mice were generated by microinjection of a 3837 bp *Sa*II–*Avr*II restriction enzyme fragment containing the CAG promoter, murine CSE cDNA, and a polyadenylation site (Fig. 1A). The mice were housed in plastic cages in a climate-controlled animal room (temperature, 24°C ± 1°C; humidity, 55% ± 5%) with a 12 h light–dark cycle (lights on at 7:00 a.m. and off at 7:00 p.m.). Mice had free access to food (Certified diet MF; Oriental Yeast, Tokyo, Japan) and water. The Animal Care and Use Committee of the University of Tsukuba approved the protocol for experiments using mice, were conducted in accordance with the Guidelines for Proper Conduct of Animal Experiments, Science Council of Japan. WT and Tg mice were fed diets supplemented with 5% CysSSCys for 2 weeks, then their tissues were harvested, organs weighed, and blood collected in tubes containing heparin via cardiac puncture. Plasma was isolated from the blood by centrifugation at 800×*g* at 4°C for 20 min. The concentration of RSS was measured in plasma and tissue samples.

### 2.3 Culture of secondary cell lines

HepG2 and HEK293 cells were obtained from the RIKEN Cell Bank (Tsukuba, Japan). They were cultured in DMEM containing 10% fetal bovine serum, 2 mM L-alanyl-l-glutamine, 100 units/mL penicillin, and 100 μg/mL streptomycin at 37°C in a humidified atmosphere with 5% CO_2_.

### 2.4 Preparation and culture of primary cells

Primary mouse hepatocytes were isolated as described previously [12,13], seeded at a density of 8 × 10^4^ cells/cm^2^ on type I collagen-coated plates (Corning Inc., NY, USA) in WME containing 10% fetal bovine serum, 2 mM L-alanyl-l-glutamine, 100 units/mL penicillin, and 100 μg/mL streptomycin, then cultured at 37°C in a humidified atmosphere with 5% CO_2_. Forty-eight hours after seeding, cells were cultured in serum-free WME for 24 h, then used for subsequent assays. Neonatal rat cardiomyocytes were isolated from 2-day-old Sprague Dawley rat pups as previously described [14], then used for subsequent assays.

### 2.5 Knockout and overexpression of SLC7A11 in HepG2 cells

SLC7A11-knockout (KO) and SLC7A11-overexpression (KOKI) HepG2 cells (Fig. 6A) were generated using CRISPR/Cas9 technology from SetsuroTech Inc (Tokushima, Japan). CRISPR/Cas9-mediated *slc7a11* gene editing was conducted following the VIKING method described previously [15] with some modifications. Briefly, genome editing of *slc7a11* gene locus and *AAVS1*, annealed oligonucleotides comprising the sequences of each gene (*slc7a11*, 5′-CACCGACAACTATAAAGAAATCTGG-3′, 5′- AAACCCAGATTTCTTTATAGTTGTC-3′, *AAVS1*, 5′-CACCGAGAGCCACATTAACCGGCCC-3′, 5′-AAACGGGCCGGTTAATGTGGCTCTC-3') were cloned into target cleaving vectors.

### 2.6 Western blotting

Mouse tissues were sonicated in lysis buffer containing 50 mM Tris-HCl (pH 7.4), 1% (v/v) Nonidet P-40, 0.1% (v/v) sodium deoxycholate, 0.1% (v/v) sodium dodecyl sulfate (SDS), 150 mM NaCl, and 1% (v/v) proteinase inhibitor cocktail. The tissue lysates were centrifuged (9,000×*g*, 10 min, 4°C) to remove insoluble material. HepG2 cells were collected by scraping into 2% SDS solution, then heated at 95°C for 15 min. Protein concentrations were determined using a bicinchoninic acid protein assay reagent kit (Pierce Biotechnology, IL, USA) before adding 2-mercaptoethanol and bromophenol blue. The protein samples (10 μg/well) were separated using SDS-PAGE and then electrotransferred onto a polyvinyl difluoride membrane (FluoreTrans; Bio-Rad Laboratories, CA, USA) at 2 mA/cm^2^ for 1 h, as described previously [16]. Membranes were blocked with 5% (w/v) skim milk in Tris-buffered saline with detergent (20 mM Tris-HCl [pH 7.5], 150 mM NaCl, and 0.1% (v/v) Tween 20) and then incubated for 1 h at room temperature with an anti-GAPDH antibody (Santa Cruz Biotechnology, CA, USA, #FL-335), anti-SLC7A11 antibody (Abcam, Cambridge, UK, #ab37185), or an anti-CSE antibody, prepared as described previously [17]. Immunoreactive bands were labeled with an enhanced chemiluminescent probe (Chemi-Lumi One L; Nacalai Tesque, Kyoto, Japan) and detected using a LAS-3000 luminescent imager (Fujifilm, Tokyo, Japan).

### 2.7 Cell viability assay

The 3-(4,5-dimethylthiazol-2-yl)-2,5-triphenyl tetrazolium bromide (MTT) assay was used to estimate cell viability, as described previously [18]. Briefly, primary mouse hepatocytes, neonatal rat cardiomyocytes, and HepG2 cells, prepared as above in 96-well plates, were treated with Na_2_S_4_ in serum-free medium and cultured at 37°C in a humidified atmosphere with 5% CO_2_. Untreated cells were used as control. After 24 h, cells were treated with 5 mg/mL MTT for 0.5 h at 37°C, then the medium was removed and 100 μL/well dimethylsulfoxide was added to dissolve the formazan precipitate. Absorbance at 540 nm was measured using an iMark microplate reader (Bio-Rad Laboratories, CA, USA).

### 2.8 Sulfur nucleophile detection

Liquid chromatography tandem mass spectrometry (LC/MS/MS) and the HPE-IAM probe was used to determine the concentration of sulfur nucleophiles, including persulfides, in the mouse brain, as previously reported [12,19]. Naive cells or those pretreated with Na_2_S_4_ for 1 h were incubated with fresh medium (serum-free medium or Hanks’ balanced salt solution [HBSS]). The medium was collected at each time point and incubated with 5 mM HPE-IAM at 37°C for 30 min to yield β-(4-hydroxyphenyl)ethyl acetamide (HPE-AM) adducts for extracellular sulfur nucleophile detection. At each time point, cells were washed with phosphate buffered saline (PBS) and collected by scraping into PBS, then pelleted by centrifugation (500×*g*, 5 min, 4°C). Using an ultrasonic disruptor (UD-201; Tomy, Tokyo, Japan), cell pellets were homogenized in 100 μL ice-cold methanol containing 1 mM HPE-IAM, then the lysates were incubated at 37°C for 30 min to yield HPE-AM adducts. Mouse tissue samples (40 mg each) were homogenized in 1 mL methanol using an ultrasonic disruptor (UD-201), then centrifuged (9,000×*g*, 10 min, 4°C) to remove insoluble material. The supernatants were incubated with 5 mM HPE-IAM at 37°C for 30 min to yield HPE-AM adducts.

To determine sulfur nucleophile concentration, aliquots containing HPE-AM adducts were diluted with 0.1% (v/v) formic acid containing known amounts of isotope-labeled internal standards, then LC/MS/MS analysis was performed using an EVOQ Qube triple quadrupole mass spectrometer (Bruker, Billerica, MA, USA) coupled to an Advance ultra-high-performance liquid chromatography system (Bruker). Sulfane sulfur-derived HPE-AM adducts were separated using the Advance system with a Triart C18 column (length, 50 mm; ID, 2.0 mm; YMC, Kyoto, Japan) at 40°C. Mobile phases A (0.1% [v/v] formic acid) and B (0.1% [v/v] formic acid in methanol) at a flow rate of 0.2 mL/min were linearly mixed using the following gradients: 3% B for 3 min; linear increase over 12 min to 95% B; constant 95% B for 1 min; linear decrease to 3% B. A heated electrospray ionization source with the following settings was used to obtain MS spectra: spray voltage, 4000 V; cone temperature, 350°C; heated probe temperature, 250°C; cone gas pressure, 25 psi; probe gas pressure, 50 psi; nebulizer gas pressure, 50 psi.

### 2.9 CSE activity measurement

Liver tissues from WT and CSE Tg mice were homogenized in 50 mM Tris-HCl (pH 7.5) containing 1% (v/v) proteinase inhibitor cocktail, then centrifuged at 9,000×*g* for 10 min at 4°C. The supernatants were applied to a PD MiniTrap G-25 column (GE Healthcare, WI, USA) equilibrated with 50 mM Tris-HCl (pH 7.5) to obtain high-molecular-weight (HMW) fractions. The reaction mixtures (100 μL) contained 100 mM HEPES buffer (pH 7.5), 1 mM cystathionine or CysSSCys, 0.1 mM pyridoxal phosphate, and 100 μg protein from the HMW fraction. They were incubated at 37°C for 30 min, followed by incubation with 5 mM HPE-IAM at 37°C for 30 min, and then deproteinized in methanol. After centrifugation at 14,000×*g* for 10 min at 4°C, the supernatants were analyzed using LC-ESI-MS/MS.

### 2.10 RNA sequencing

Using an RNeasy Lipid Tissue Mini Kit (Qiagen, Hilden, Germany), total RNA was extracted from WT primary mouse hepatocytes treated with or without 100 μM Na_2_S_4_ for 12 h. The concentration and quality of the RNA were determined using a Nanodrop system (Thermo Fisher Scientific, CA, USA) and a Bioanalyzer RNA 6000 Pico Kit (Agilent technologies, CA, USA), respectively. Total RNA (500 ng) was subjected to rRNA depletion and subsequent library synthesis using a NEBNext rRNA Depletion Kit and Ultra Directional RNA Library Prep Kit (New England Biolabs, MA, USA), respectively. Then, 2 × 36 paired-end sequencing was performed using a NextSeq500 sequencer (Illumina, CA, USA) by Tsukuba i-Laboratory LLP (Tsukuba, Japan). FASTQ files were imported to the CLC Genomics Workbench software (Qiagen, Venlo, Netherlands), sequences mapped to the mm10 mouse genome, and annotated genes in the ENSEMBLE database quantified. Up-regulation was defined as a log2-fold increase in relative transcription levels or greater.

### 2.11 Real-time polymerase chain reaction

Total RNA from WT or nuclear factor-erythroid factor 2-related factor 2 (Nrf2) knockout (KO) primary mouse hepatocytes treated with or without 100 μM Na_2_S_4_ for 1 h was extracted using an RNeasy Lipid Tissue Mini Kit, and cDNA was synthesized using a High-Capacity cDNA Reverse Transcription Kit (Applied Biosystems, CA, USA), following the manufacturers’ protocols. Real-time PCR was performed using Power SYBR Green PCR Master Mix (Applied Biosystems) with a 7500 Real-Time PCR System (Applied Biosystems). The following PCR primers were used: forward 5′-GTCATCGGATCAGGCATCTT-3′ and reverse 5′-CATAGGACAGGGCTCCAAAA-3′ for *Slc7a11*, and forward 5′-GGAGAATGGGAAGCCGAACA-3′ and reverse 5′-TCCTTGCTGAAGGACATATCTGACA -3′ for β2-microglobulin (*B2m*). The PCR conditions were as follows: 50°C for 2 min, 95°C for 10 min, and 45 cycles of 95°C for 15 s and 60°C for 1 min. Melting curve analysis was conducted to ensure amplification of a single product. The *Slc7a11* and *B2m* mRNA levels in each RNA sample were determined using the relative standard curve method. Changes in *Slc7a11* expression were assessed after the fluorescence intensity of its PCR product had been normalized relative to that of *B2m*.

### 2.12 Protein persulfidation measurement

Iodoacetyl-biotin was used to analyze protein persulfidation as previously described [20], with some modifications. Primary cardiomyocytes and hepatocytes were treated with or without 100 μM Na_2_S_4_ for 1 h, then incubated in control medium for an additional 3 h HepG2 cells were treated with or without 400 μM Na_2_S_4_ for 1 h, then incubated in HBSS for an additional 1 h. Cells were washed with ice-cold PBS and collected in lysis buffer (40 mM phosphate [pH 7.4], 150 mM NaCl, 1% [v/v] Triton X-100, 0.1% [v/v] SDS, and 3 mM tyrosine) containing aprotinin (2 μg/ml) and pepstatin A (10 μg/ml). The cell lysate was centrifuged (16,000×*g*, 10 min, 4°C), and the protein concentration of supernatant was adjusted to 1.5 mg/mL. The sample was incubated with 500 μM iodoacetyl-PEG_2_-biotin (Thermo Fisher Scientific, CA, USA) at 37°C for 30 min to label thiol groups, and methanol/chloroform precipitation was used to remove the unbound label. The resuspended sample was incubated with control agarose beads (Thermo Fisher Scientific, CA, USA) at 4°C for 2 h to remove non-specific binding proteins. The biotinylated proteins were pulled down using NeutrAvidin high-capacity agarose beads (Thermo Fisher Scientific, CA, USA) at 4°C for 5 h. The beads were washed three times with lysis buffer and twice with lysis buffer without tyrosine. Persulfidated proteins were eluted using 40 mM dithiothreitol in lysis buffer without tyrosine. Eluted samples were mixed with Laemmli buffer and subjected to SDS-PAGE. Proteins were visualized using Flamingo Fluorescent Protein Gel Stain (Bio-Rad Laboratories, CA, USA).

### 2.13 Mitochondrial membrane potential measurement

Primary cardiomyocytes and hepatocytes were plated on a glass bottom dish (AGC Techno Glass, Tokyo, Japan) and type I collagen-coated glass-bottom chamber (Matsunami Glass Ind., Tokyo, Japan), respectively. Cells were treated with 2 μM JC-1 (Thermo Fisher Scientific, CA, USA) and 2 μM Hoechst 33342 (Nacalai Tesque Inc., Kyoto, Japan) in HBSS for 30 min, washed twice with FluoroBrite DMEM (Thermo Fisher Scientific, CA, USA), then incubated with FluoroBrite DMEM with or without Na_2_S_4_ for 45 min. The JC-1 and Hoechst 33342 signals were measured using a BZ-X700 fluorescence microscope (Keyence, Osaka, Japan) and the red/green ratio of the acquired images was quantified.

### 2.14 Quantitation of creatine kinase and alanine aminotransferase in plasma

Plasma concentrations of creatine kinase and alanine aminotransferase were measured using a dry-chemistry analyzer (DRI-CHEM NX500, Fuji Film, Tokyo, Japan), following the manufacturer's instructions.

### 2.15 Echocardiography analysis

Echocardiography was performed using a high-frequency ultrasound imaging system (Prospect T1, Scintica) with mice under isoflurane anesthesia (induction: 2.0%–2.5%), as described previously [21].

### 2.16 Statistical analysis

Statistical analyses were performed using Student's unpaired *t*-test, one-way ANOVA, two-way ANOVA, or two-way repeated-measures ANOVA, as appropriate, and followed by post hoc testing recommended by the GraphPad Prism software (v. 9.2.0; GraphPad Software, Inc., CA, USA). A *p* value of less than 0.05 was considered statistically significant.

## 3 Results

### 3.1 Alterations in intracellular and extracellular concentrations of persulfides in WT and CSE Tg mice

We generated CSE Tg mice using the vector shown in Fig. 1A. Western blot analysis of CSE in a variety of tissues from WT and CSE Tg mice (Fig. 1B and C) revealed that the protein was constitutively expressed in liver and kidney of WT mice. In addition, CSE expression levels in the heart and skeletal muscles were significantly higher in Tg mice than in WT mice (Fig. S1). Because CSE catalyzes transformation of cystathionine to CysSH and of CysSSCys to CysSSH [6,11], we measured the CSE enzyme activity in Tg and WT mice. As shown in Fig. 1D and E, enzymatic production of CysSH from cystathionine by the HMW fraction of liver from CSE Tg mice was significantly greater than that of the corresponding fraction from WT mice. When CysSSCys was used as a substrate, formation of CysSSH and its polysulfide CysSSSH was significantly enhanced by overexpression of CSE. Similar results were also obtained for heart tissue from WT and CSE Tg mice (Fig. S2). However, the concentrations of CysSH, GSH, and their persulfides in liver, kidney, and heart of WT mice were not markedly different from their respective concentrations in CSE Tg mice, except for CysSH in the heart and GSSH in kidney (Figs. S3 and 1F). In contrast, plasma concentrations of CysSSH and GSSH were significantly higher in CSE Tg mice compared with WT (Fig. 1F and G). We therefore speculated that CysSSH and GSSH overproduced by overexpression of CSE could be exported to extracellular space.

### 3.2 Excess intracellular persulfides are exported into extracellular space

**Fig. 2:**
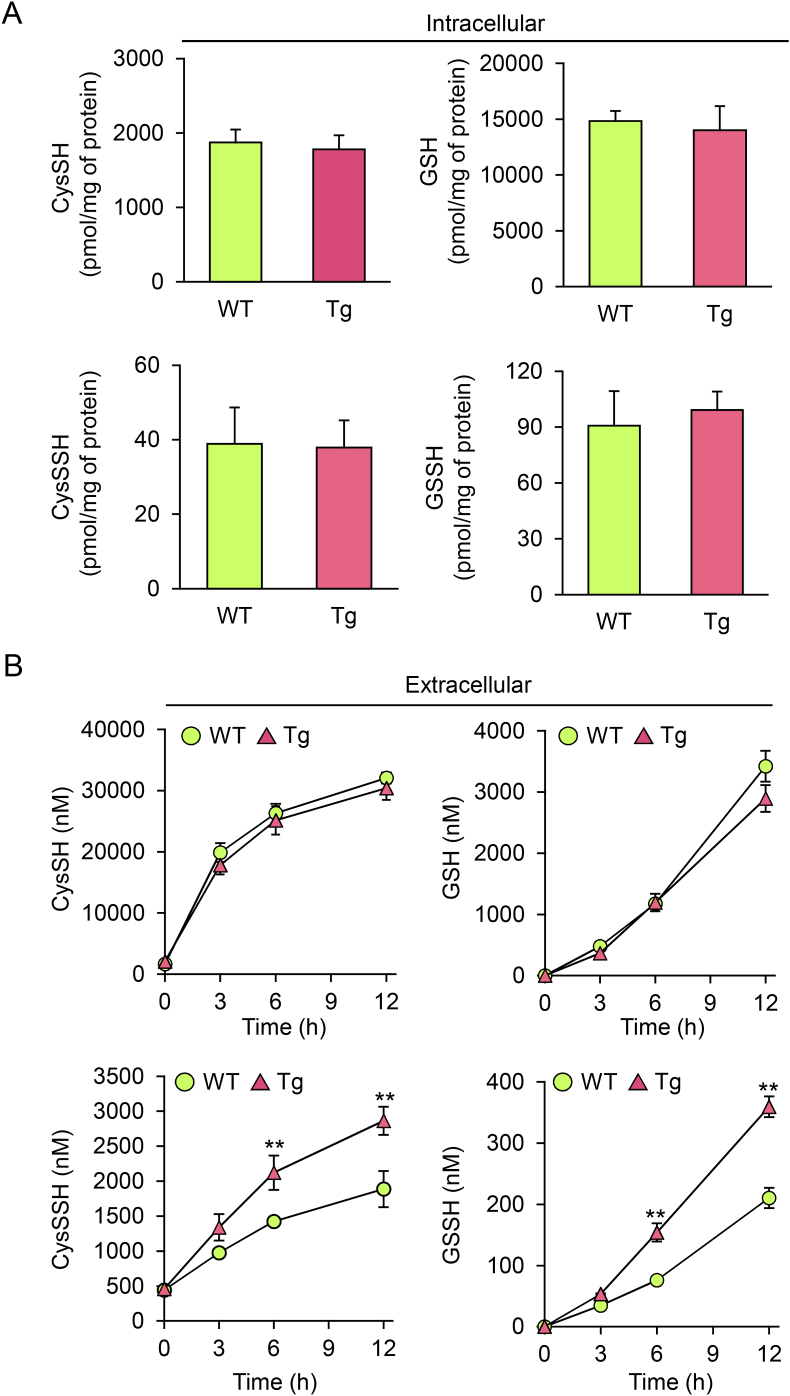
Effect of CSE overexpression on intra- and extra-cellular concentrations of sulfur nucleophiles in primary hepatocytes. (A) Intracellular and (B) extracellular concentrations of CysSH, GSH, CysSSH, and GSSH produced by hepatocytes incubated in serum-free Williams' Medium E (WME) were quantitated using LC-ESI-MS/MS. Concentrations in (A) were obtained from sonicated cell lysate after 12 h incubation and those in (B) were obtained from media fractions collected at the indicated times. Data represent the mean ± SEM (*n* = 3). **p < 0.01.

To address the above hypothesis, we prepared primary hepatocytes from WT and CSE Tg mice and determined intracellular and extracellular concentrations of CysSH, GSH, and their persulfides (Fig. 2). Consistent with the *in vivo* tissue analyses (Fig. 1), intracellular thiol concentrations in primary hepatocytes from WT mice were almost the same as those from CSE Tg mice (Fig. 2A). However, time-dependent concentrations of CysSSH and GSSH, but not CysSH and GSH, in the culture medium of primary hepatocytes from CSE Tg mice were significantly greater than those from WT mice (Fig. 2B). This suggests that persulfides such as CysSSH and GSSH, excessively produced by overexpression of CSE, are excreted into the extracellular space from primary hepatocytes.

**Fig. 3:**
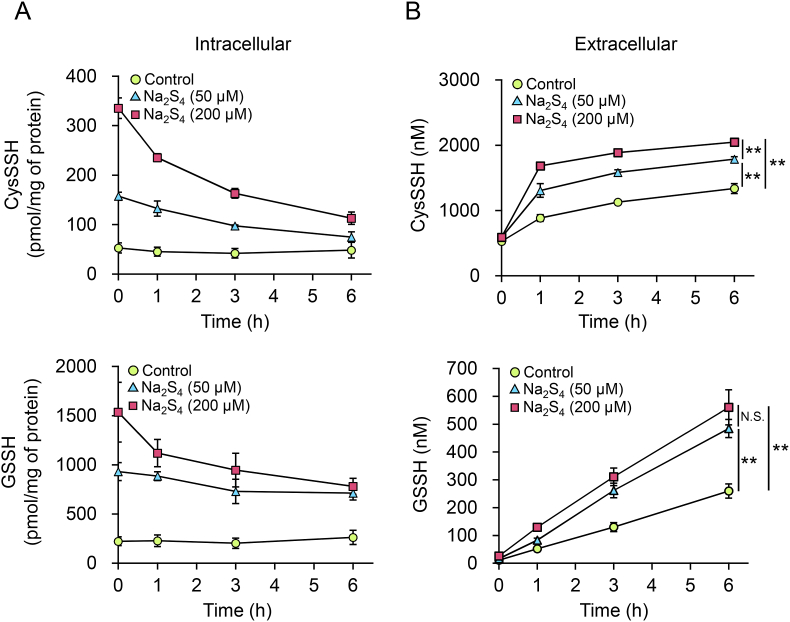
Effects of Na_2_S_4_ on intra- and extra-cellular concentrations of persulfides in primary hepatocytes. (A and B) Cells were treated with serum-free WME containing 0 (control), 50, or 200 μM Na_2_S_4_ for 1 h, washed three times, and incubated in fresh serum-free WME. At the indicated times, (A) cells and (B) medium were collected and their persulfide concentrations quantified using LC-ESI-MS/MS. Data represent the mean ± SEM (*n* = 3). **p < 0.01, N.S.: not significant.

To identify an alternative to using CSE Tg mice, we incubated Na_2_S_4_ with either CysSH or CysSSCys in a cell-free system and assessed its ability to induce excessive RSS production. Interaction of Na_2_S, a generator of hydrogen sulfide, with CysSSCys resulted in production of CysSSH (Table 1). When Na_2_S_4_ was used instead of Na_2_S, formation of CysSSH was markedly increased and CysSSSH was also formed. Therefore, we exposed primary mouse hepatocytes from WT mice to Na_2_S and Na_2_S_4_ to induce excess intracellular RSS. The intra- and extra-cellular concentrations of CysSH and GSH were the same before and after exposure to Na_2_S_4_ (Fig. S4). The intracellular concentrations of persulfides CysSSH and GSSH rapidly increased upon Na_2_S_4_ exposure and then slowly declined with their excretion into the extracellular space (Fig. 3). In contrast, Na_2_S exposure produced minimal changes in CysSH, GSH, CysSSH and GSSH (Fig. S5). Alterations in intra- and extra-cellular persulfide concentrations in response to Na_2_S_4_ exposure were also observed in primary cultured astrocytes (Fig. S6).

**Table 1:** Cysteine persulfide (CysSSH) and cysteine polysulfide (CysSSSH) generated by the reaction of cysteine (CysSH) or cystine (CysSSCys) with sulfur donors.

	Na_2_S	CysSH (μM)	CysSSH (μM)	CysSSSH (μM)
100 μM CysSH	–	74.8 ± 3.5	N.D.	N.D.
	+	74.1 ± 2.5	2.2 ± 0.3	N.D.
100 μM CysSSCys	–	N.D.	N.D.	N.D.
	+	15.6 ± 0.1	22.2 ± 1.2	N.D.
	Na_2_S_4_	CysSH (μM)	CysSSH (μM)	CysSSSH (μM)

100 μM CysSH	–	78.1 ± 1.8	2.0 ± 1.7	N.D.
	+	7.9 ± 1.4	64.7 ± 0.7	34.3 ± 1.1
100 μM CysSSCys	–	N.D.	N.D.	N.D.
	+	7.3 ± 0.6	65.7 ± 7.6	41.8 ± 3.7

Note: One-hundred micromolar Na_2_S or Na_2_S_4_ was incubated with 100 μM CysSH or CysSSCys in 50 mM HEPES (pH 7.5) at 37°C for 30 min. The reaction mixture was incubated with 1 mM β-(4-hydroxyphenyl)ethyl (HPE) iodoacetamide at 37°C for 30 min to obtain HPE acetamide adducts, which were identified using LC/MS/MS and quantified using isotope-labeled internal standards. N.D., not detected.

### 3.3 Identification of antiporters excreting CysSSH into the extracellular space

**Fig. 4:**
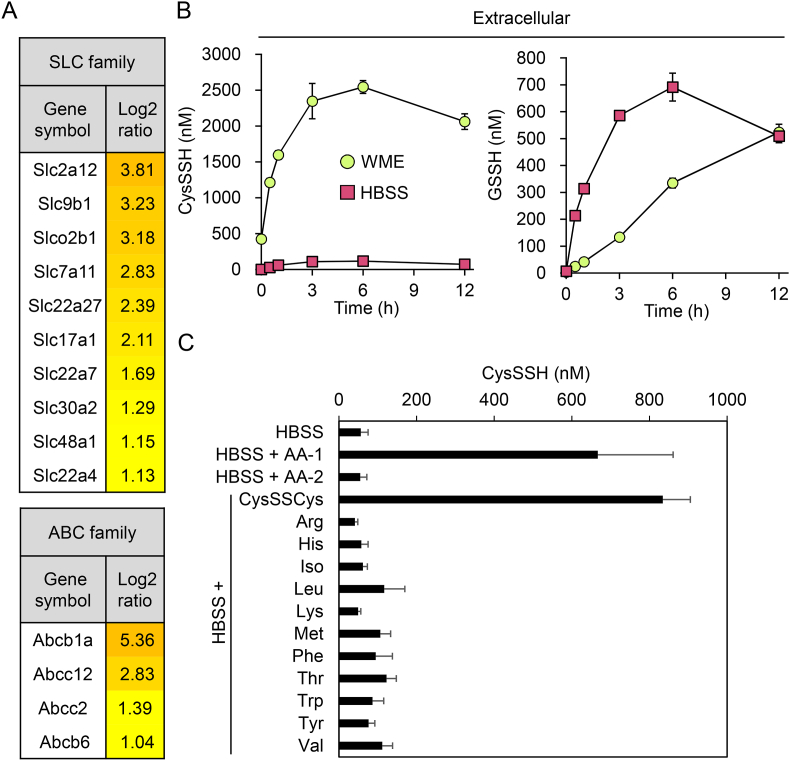
Amino acid requirements for export of CysSSH in primary mouse hepatocytes. (A) Gene expression of numerous SLC and ABC transporter isoforms in hepatocytes treated with or without 100 μM Na_2_S_4_ for 12 h in serum-free WME. The expression levels of SLC and ABC family genes upregulated upon Na_2_S_4_ treatment are expressed as log2-fold increases relative to the untreated control. (B) Concentrations of extracellular CysSSH and GSSH produced by hepatocytes in serum-free WME (with amino acids) and Hanks' balanced salt solution (HBSS; without amino acids) treated with 100 μM Na_2_S_4_ for 1 h. Following treatment, cells were washed three times and incubated in serum-free WME or HBSS, then media were collected at the indicated time points and their CysSSH and GSSH concentrations quantified using LC-ESI-MS/MS. (C) Concentrations of extracellular CysSSH produced by hepatocytes treated with 100 μM Na_2_S_4_ for 1 h and incubated in HBSS supplemented with individual amino acids for 3 h. The conditioned culture medium was collected and the CysSSH concentration quantified using LC-ESI-MS/MS. AA-1, MEM essential amino acids solution; AA-2, MEM non-essential amino acids solution; CysSSCys (100 μM), cystine; Arg (600 μM), arginine; His (200 μM), histidine; Iso (400 μM), Isoleucine; Leu (400 μM), leucine; Lys (400 μM), lysine; Met (100 μM), methionine; Phe (200 μM), phenylalanine; Thr (400 μM), threonine; Try (50 μM), tryptophan; Tyr (200 μM), tyrosine; Val (400 μM), valine. Data represent the mean ± SEM (*n* = 3).

RNA sequencing revealed that gene expression of numerous SLC and ATP-binding cassette (ABC) transporter isoforms in primary mouse hepatocytes was increased upon Na_2_S_4_ exposure (Fig. 4A), suggesting that these transporters may participate in persulfide export. While GSH is excreted via ABC transporters [22], MK571, which is an inhibitor of ABCC transporters [23], did not affect export of CysSSH and unexpectedly enhanced export of GSSH (Fig. S7). This suggests that ABCC transporters are unlikely to be involved in CysSSH export and that transporters responsible for export of CysSSH and GSSH are distinct. Culture of primary mouse hepatocytes in media with (WME) and without (HBSS) amino acids suggested that transporters for CysSSH export require amino acids, whereas those for GSSH export do not (Fig. 4B). We supplemented the HBSS medium with individual amino acids and found that CysSSCys is indeed required for export of CysSSH from the cells (Fig. 4C). This requirement for CysSSCys was also observed using HepG2 cells (Fig. S8).

**Fig. 5:**
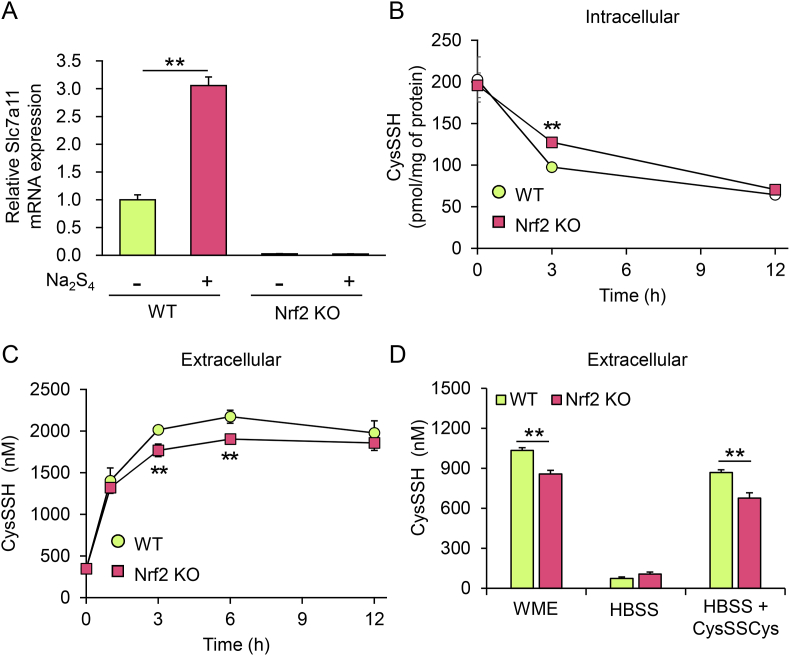
Effect of Nrf2 deletion on intracellular and extracellular CysSSH transitions. (A) *Slc7a1*1 mRNA levels determined using real-time PCR and expressed relative to those in the untreated WT cells. Data represent the mean ± SEM (*n* = 3). **p < 0.01. (B) Intracellular and (C) extracellular concentrations of CysSSH produced by hepatocytes treated with 100 μM Na_2_S_4_ for 1 h in serum-free WME. Following treatment, cells were washed three times and incubated in serum-free WME, then cells and media were collected at the indicated time points and their CysSSH concentrations quantified using LC-ESI-MS/MS. Data represent the mean ± SEM (*n* = 3). **p < 0.01. (D) Extracellular CysSSH concentration after treating hepatocytes with serum-free WME containing 100 μM Na_2_S_4_ for 1 h. Following treatment, cells were washed three times and incubated in WME, HBSS, or HBSS +100 μM CysSSCys for 3 h, then the conditioned culture media were collected and CysSSH concentrations quantified using LC-ESI-MS/MS. Data represent the mean ± SEM (*n* = 3). **p < 0.01.

SLC7A11 was reported to be an antiporter for CysSSCys and glutamate [24], and therefore we investigated the contribution of SLC7A11 to export of CysSSH from primary hepatocytes. The mRNA level of *Slc7a11* was markedly increased upon Na_2_S_4_ exposure of primary hepatocytes from WT mice, whereas there was no induction of *Slc7a11* expression during Na_2_S_4_ exposure of primary hepatocytes from Nrf2 KO mice (Fig. 5A) because this transcription factor regulates *Slc7a11* expression [25]. The Na_2_S_4_-dependent decrease in intracellular CysSSH concentration (Fig. 5B) and increase in extracellular CysSSH concentration (Fig. 5C) in primary hepatocytes from WT mice were slightly but significantly greater than those measured using primary hepatocytes from Nrf2 KO mice. Such a moderate effect of Nrf2 deletion on CysSSH export was also seen in experiments using either WME or HBSS in the presence of CysSSCys (Fig. 5D). The requirement of CysSSCys for export of CysSSH was also confirmed using HepG2 and HEK293 cells, as was the blockage of CysSSH excretion into the extracellular space by sulfasalazine, a specific inhibitor of SLC7A11 [26] (Fig. S9). These results suggest that SLC7A11 partially participates in CysSSH export from primary hepatocytes.

### 3.4 Role of SLC7A11 as a CysSSH/CysSSCys antiporter in HepG2 cells

**Fig. 6:**
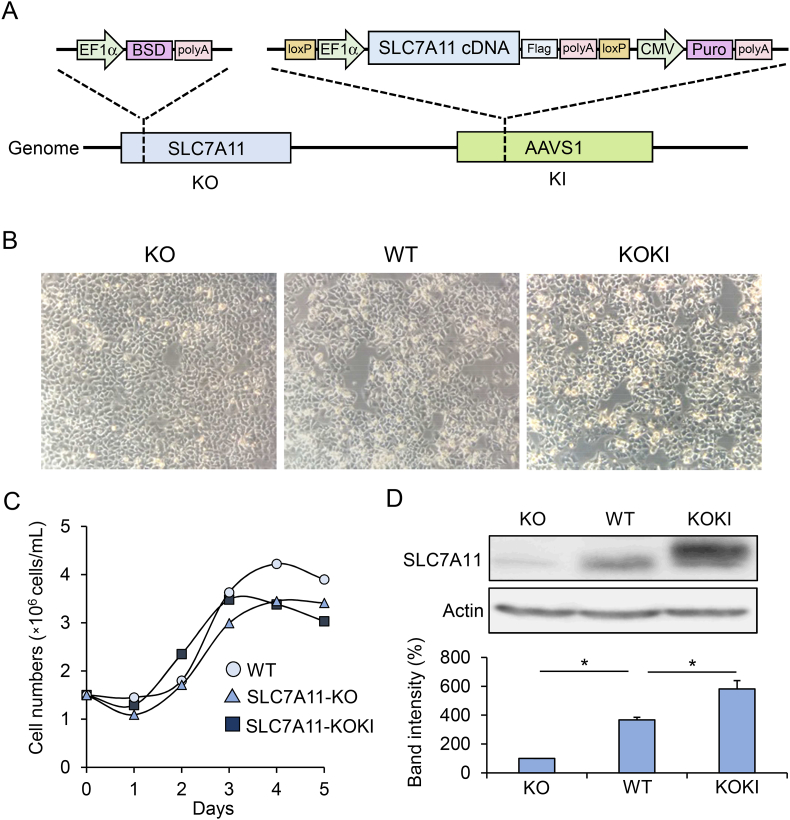
Properties of HepG2 cells with KO and overexpression (KOKI) of SLC7A11. (A) Schematic drawing of CRISPR/Cas9-mediated genome editing. (B) Cell morphologies. (C) Cell proliferation. (D) Western blot analysis of SLC7A11 protein concentrations in cell lysates. Bands were quantified using ImageJ software and data represent the mean ± SD (*n* = 3). *p < 0.05.

**Fig. 7:**
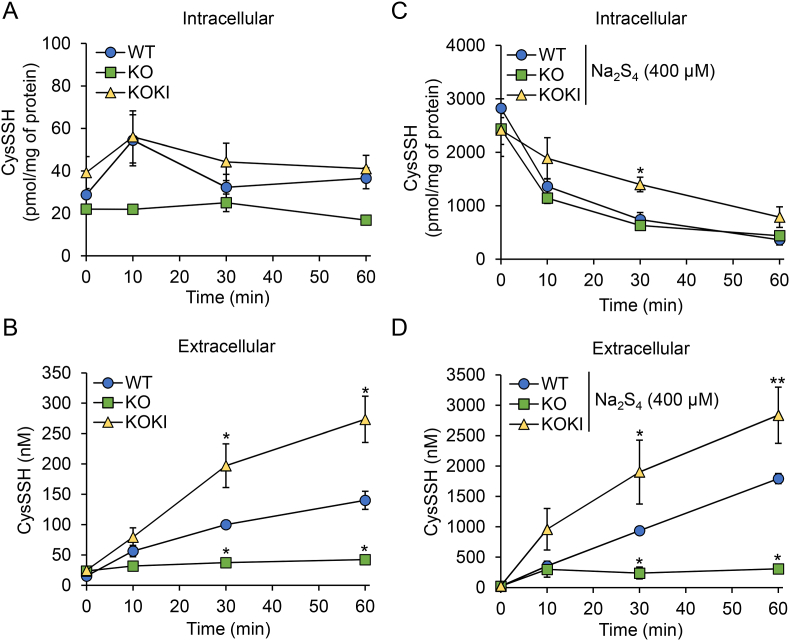
SLC7A11 is an CysSSH exporter in HepG2 cells. (A) Intracellular and (B) extracellular concentrations of CysSSH from WT, SLC7A11-KO, and SLC7A11-KOKI cells cultured in HBSS containing 100 μM CysSSCys. Cells and media were collected at the indicated time points and their CysSSH concentrations quantified using LC-ESI-MS/MS Data represent the mean ± SEM (*n* = 3). *p < 0.05, compared with WT. (C) Intracellular and (D) extracellular concentrations of CysSSH from WT, SLC7A11-KO, and SLC7A11-KOKI cells treated with 400 μM Na_2_S_4_ in DMEM for 1 h. After treatment, cells were washed three times and incubated with HBSS containing 100 μM CysSSCys, then cells and media were collected at the indicated time points and their CysSSH concentrations quantified using LC-ESI-MS/MS. Data represent the mean ± SE (*n* = 3). *p < 0.05, **p < 0.01, compared with WT.

The inhibitory effect of sulfasalazine on export of CysSSH from HepG2 cells (Fig. S9) indicates that SLC7A11 is an important antiporter for CysSSH export. To clarify its role in CysSSH excretion into the extracellular space, we established HepG2 cell lines with SLC7A11 KO and SLC7A11 KOKI (Fig. 6A). There were no obvious differences in cell morphologies (Fig. 6B) or numbers (Fig. 6C) among WT, SLC7A11 KO, and SLC7A11 KOKI HepG2 cells. Minimal expression of SLC7A11 protein was detected in KO cells, and overexpression was confirmed in KOKI cells (Fig. 6D). Using these cells, we measured the time-dependent intra- and extra-cellular concentrations of CysSSH. When HBSS containing CysSSCys was used as a growth medium, no differences in intracellular CysSSH concentrations were measured among WT, SLC7A11 KO, and SLC7A11 KOKI cells; however, the concentration of CysSSH excreted from the KO and KOKI cells was significantly lower and higher, respectively, than that excreted from WT cells (Fig. 7A and B). When exposed to 400 μM Na_2_S_4_, the export of CysSSH from KOKI cells was marked and that from KO cells was negligible, although the time-dependent decrease of intracellular CysSSH concentration in KOKI cells was unexpectedly slower than that in the WT and KO cells (Fig. 7C and D). The requirement of SLC7A11 for extracellular efflux of excess intracellular CysSSH was also confirmed by treatment with CysSSSCys (Fig. S10), an alternate RSS donor [27].

**Fig. 8:**
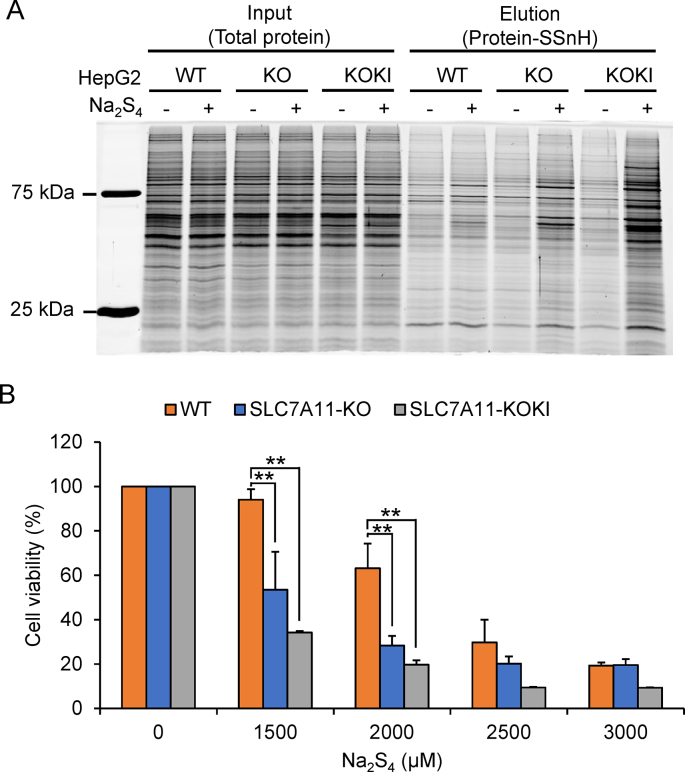
SLC7A11 regulates excess RSS-induced stress in HepG2 cells. (A) SDS-PAGE stained with Flamingo fluorescent gel stain analysis of per- and poly-sulfidated proteins (protein-SSnH) in WT and SLC7A11-KO HepG2 cells following treatment with (+) or without (−) 400 μM Na_2_S_4_ for 1 h and incubated for an additional 1 h in HBSS. (B) MTT assay of WT and SLC7A11-KO HepG2 cell viability after exposure to variable Na_2_S_4_ concentrations for 24 h. Data represent the mean ± SEM (*n* = 3). **p < 0.01.

Previous studies have postulated that cellular proteins can undergo polysulfidation, either by excessive production of CysSSH in response to Na_2_S_4_ exposure (see Table 1) or by Na_2_S_4_ itself [2,28]. To address this issue, we examined alterations in polysulfidated proteins in SLC7A11 KO and SLC7A11 KOKI cells during Na_2_S_4_ exposure. As shown in Fig. 8A, the amount of per- and poly-sulfidated protein (protein-SSnH) in KO cells was greater than that in WT cells and less than that in KOKI cells which exhibit a slow decay of intracellular CysSSH concentration (Fig. 7C). Interestingly, the relative amount of protein-SSnH in each cell line (KOKI > KO > WT) was consistent with the concentration-dependent Na_2_S_4_ cytotoxicity (Fig. 8B).

### 3.5 Differential sensitivity of hepatocytes and cardiomyocytes to intracellular excess RSS

**Fig. 9:**
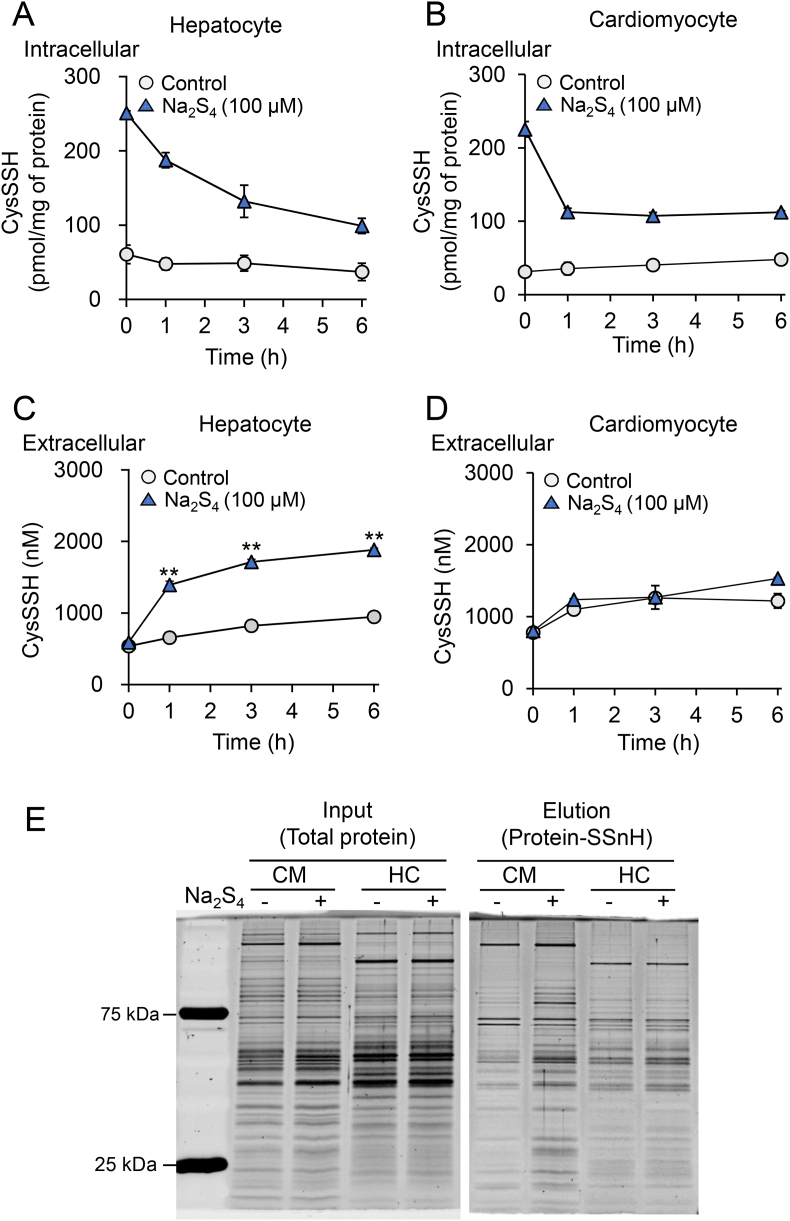
Limited excretion of excess RSS causes the accumulation of persulfidated proteins in cardiomyocytes. (A,B) Intracellular and (C,D) extracellular CysSSH concentrations produced by (A,C) primary hepatocytes and (B,D) primary cardiomyocytes prepared from WT mice, following treatment with 100 μM Na_2_S_4_ in serum-free medium for 1 h. After treatment, cells were washed three times and incubated in serum-free medium, then cells and media were collected at the indicated time points and their CysSSH concentrations quantified using LC-ESI-MS/MS. (E) SDS-PAGE stained with Flamingo fluorescent gel stain analysis of protein-SSnH in primary cardiomyocytes (CM) and hepatocytes (HC) treated with (+) or without (−) 100 μM Na_2_S_4_ for 1 h and incubated for an additional 3 h in serum-free medium. Data represent the mean ± SEM (*n* = 3). **p < 0.01.

**Fig. 10:**
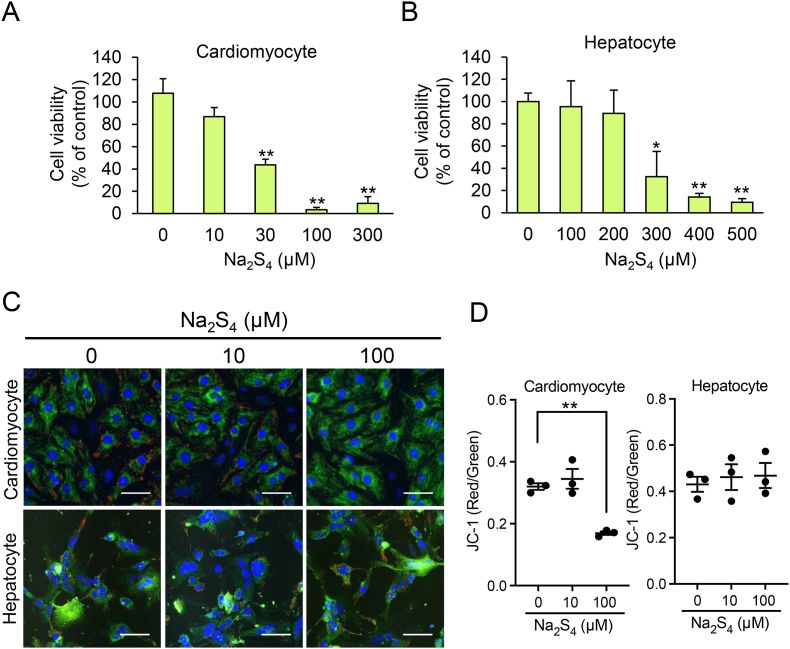
Differential sensitivity of hepatocytes and cardiomyocytes to excess RSS. The viability of (A) primary cardiomyocytes and (B) hepatocytes after treatment with variable Na_2_S_4_ concentrations in serum-free medium for 24 h was determined using an MTT assay. Data represent the mean ± SEM (*n* = 3). *p < 0.05, **p < 0.01, compared with Na_2_S_4_ untreated sample (0 μM). (C) Representative fluorescence microscopy images and (D) quantitation of the average ratio of red/green fluorescence of primary cardiomyocytes and hepatocytes loaded with the JC-1 probe of mitochondrial membrane potential, then treated with variable Na_2_S_4_ concentrations for 45 min. Blue: Hoechst 33342 (nucleus). Red/green: JC-1. Scale bars: 50 μm. Data represent the mean ± SEM (*n* = 3). **p < 0.01. (For interpretation of the references to colour in this figure legend, the reader is referred to the Web version of this article.)

To examine whether the efflux of CysSSH and sensitivity to excess intracellular CysSSH differed between cell types, we measured the cell viability and intra- and extracellular concentrations of CysSSH and protein-SSnH following exposure of hepatocytes and cardiomyocytes to 100 μM Na_2_S_4_. Unlike hepatocytes, the intracellular concentration of CysSSH in cardiomyocytes decreased to a plateau after 1 h of Na_2_S_4_ exposure (Fig. 9A and B) and a negligible increase in their extracellular CysSSH concentration was measured (Fig. 9C and D). Under these conditions, the protein-SSnH concentration in cardiomyocytes was greater than that in hepatocytes (Fig. 9E). In cardiomyocytes, a significant cytotoxicity was measured at a Na_2_S_4_ concentration of 30 μM (Fig. 10A). In contrast, the cell viability of hepatocytes was unaffected even at 200 μM Na_2_S_4_ (Fig. 10B). Compared with untreated cells, the mitochondrial membrane potential, measured using the probe JC-1, was significantly reduced following treatment with 100 μM Na_2_S_4_ for 45 min in cardiomyocytes but not hepatocytes (Fig. 10C and D), the mitochondrial membrane potential of the latter being significantly reduced by treatment with 500 μM Na_2_S_4_ (Fig. S11).

### 3.6 Effects of abnormal RSS production caused by excessive CysSSCys administration on CSE Tg mice

**Fig. 11:**
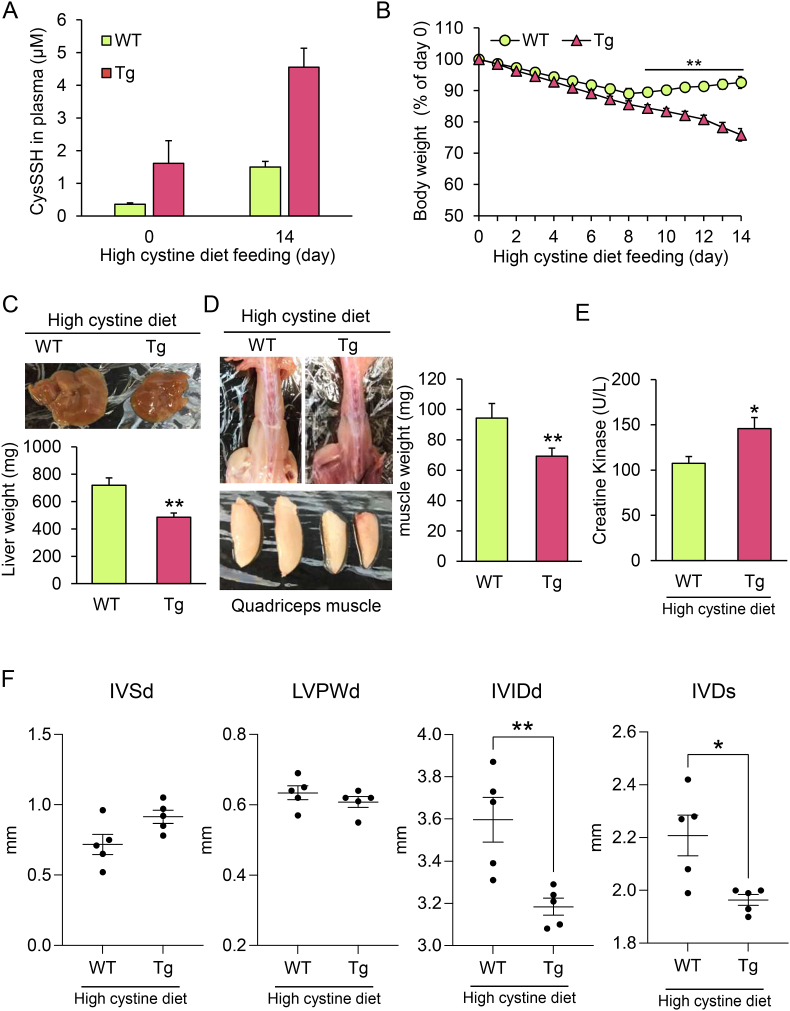
Effects of excessive CysSSCys administration on CSE Tg mice. WT and Tg mice were fed a diet supplemented with 5% CysSSCys for 2 weeks. (A) Plasma CysSSH concentrations (*n* = 3, each group). (B) Body weight change. Data show the mean ± SEM (*n* = 5, each genotype).*p < 0.05. (C,D) Stereoscopic images and weights of (C) liver and (D) quadricep muscle. Data represent the mean ± SEM (*n* = 3, each genotype). *p < 0.05. (E) Plasma creatine kinase (CK) concentrations. Data represent the mean ± SEM (*n* = 5, each genotype). *p < 0.05. (F) The interventricular septum diastolic thickness (IVSd), left ventricle posterior wall diastolic thickness (LVPWd), left ventricle internal diameter at end-diastole (LVIDd), and left ventricle internal diameter at end-systole (LVIDs) were measured using echocardiography. Data represent the mean ± SEM (*n* = 5, each genotype). *p < 0.05, **p < 0.01.

To induce excess RSS-induced stress *in vivo*, WT and CSE Tg mice were fed diets supplemented with an excessive amount of CysSSCys for several weeks. We observed an aberrant increase in CysSSH, but not CysSH, GSH, and GSSH, concentrations in CSE Tg mice two weeks after CysSSCy treatment (Fig. 11A and S12). The average weight of CSE Tg mice 10 d after initiating CysSSCys supplementation was significantly lower in than that of WT mice (Fig. 11B). The wet tissue weights of the liver (Fig. 11C) and quadricep muscles (Fig. 11D) were also markedly lower in CSE Tg mice fed a high-CysSSCys diet than in WT mice fed the same diet, while there were no differences between the tissue weights of WT and CSE Tg mice fed a regular diet (Fig. S13). After being fed a high-CysSSCys diet for two weeks, the plasma concentrations of creatine kinase, a blood marker of skeletal muscle damage, were significantly higher in CSE Tg mice than in WT mice (Fig. 11E), whereas their plasma concentrations of alanine aminotransferase, a blood marker of liver damage, were not significantly different (Fig. S14). Furthermore, echocardiography revealed a significantly smaller left ventricle internal diameter at end-diastole and end-systole in CSE Tg mice than in WT mice (Fig. 11F), although there were no significant differences in interventricular septum diastolic thickness, left ventricle posterior wall diastolic thickness, ejection fraction, fractional shortening, and heart rate between WT and CSE Tg mice (Fig. 11F and S15). Unlike WT mice, CSE Tg mice fed a high-CysSSCys diet continued to lose weight and eventually died (Fig. S16).

## 4 Discussion

Using CSE overexpression or treatment with Na_2_S_4_ to induce an increase in intracellular CysSSH concentration, we showed that CysSSH levels are strictly regulated via CysSSH export from tissues and cells *in vivo* and *in vitro*, suggesting an adaptive response to excess intracellular CysSSH. In the present study, a surprising discovery was that CysSSCys-dependent antiporters, including SLC7A11 participate in export of excess intracellular CysSSH. Bannai and co-workers originally demonstrated that SLC7A11, then known as xCT, is an antiporter for extracellular CysSSCys and intracellular glutamate [24]. However, it was subsequently reported that cystathionine, which is structurally similar to CysSSCys, is also an extracellular SLC7A11 substrate [29].

The present study confirmed previous findings of Cao et al. that Na_2_S_4_ activates Nrf2 [30]. Our RNA sequencing analysis revealed that expression of genes encoding SLC and ABC transporters is also up-regulated, suggesting that these transporters play a critical role in pumping CysSSH into the extracellular space. Pretreatment with a specific ABCC transporter inhibitor, MK571, did not affect transport of CysSSH, which excludes a role for ABC transporters in CysSSH excretion. However, culture of primary mouse hepatocytes using HBSS supplemented with individual amino acids clearly demonstrated that CysSSCys is essential for CysSSH export. This surprising finding was also confirmed using HepG2 and HEK293 cell lines. Induction of SLC7A11, evaluated using RNA sequencing, and suppression of CysSSH export using sulfasalazine, an SLC7A11 inhibitor, suggest that SLC7A11 participates in excretion of CysSSH into the extracellular space. In contrast, deletion of Nrf2 in primary mouse hepatocytes slightly repressed export of intracellular CysSSH. Further study is required to elucidate predominant antiporters other than SLC7A11.

**Fig. 12:**
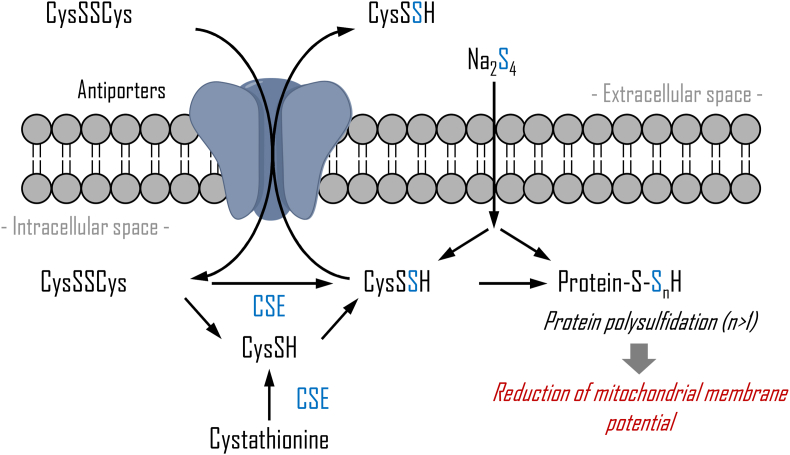
Schematic representation of the CysSSCys/CysSH/CysSSH cycle through cystine-dependent antiporters to buffer sulfur stress.

Our preliminary experiment using HepG2 cells overexpressing CSE and stable isotope-labeled CysSSCys showed that CysSSCys incorporated into the intracellular space via a CysSSCys-dependent antiporter is rapidly reduced to CysSH by a reductase such as 14 kDa thioredoxin-related protein [31], while CysSH is also produced by CSE [11]. There are at least three explanations for the enhanced intracellular CysSSH concentration as shown in Fig. 12. First, exposure to Na_2_S_4_ results in substantial CysSSH formation during its intracellular interaction with either CysSSCys or CysSH, as shown in Table 1. Second, although CysSSCys is not a good CSE substrate because of its higher Michaelis constant [32] under normal conditions, oxidation of CysSH should yield abundant intracellular CysSSCys. As a result, increased CysSSCys, in addition to overexpression of CSE, is associated with production of CysSSH. Third, because CysSH is a substrate of cysteinyl-tRNA synthetase [19], an elevated CysSH concentration potentially promotes intracellular CysSSH production. Importantly, Na_2_S_4_ exposure-induced increase in CysSSH concentration results in greater protein polysulfidation in HepG2 cells and primary cardiomyocytes, leading to a lower mitochondrial membrane potential and a concentration-dependent cytotoxicity (Fig. 10). As shown in Fig. 7C and D, despite significantly greater excretion of CysSSH from SLC7A11 KOKI cells into the extracellular space compared with that from WT or SCL7A11 KO cells, the time-dependent decrease of intracellular CysSSH in KOKI cells was slower than that observed in WT and KO cells. This may be because SLC3A2, which is a key partner protein required for maximal activity of SLC7A11 [24], was not co-overexpressed with SLC7A11 in the present study.

Post-translational protein modifications produced by RSS are associated with redox homeostasis [2,3]. In the present study, however, excess intracellular RSS by Na_2_S_4_ exposure in HepG2 cells, primary mouse hepatocytes, and rat primary cardiomyocytes caused 1) an increase in the concentrations of polysulfidated proteins, 2) a decrease in the mitochondrial membrane potential, and 3) cytotoxicity. Interestingly, SLC7A11 KO HepG2 cells and primary cardiomyocytes, which excrete limited quantities of CysSSH, were more sensitive to excess intracellular RSS-induced stress because of the greater degree of polysulfidation of intracellular proteins compared to WT HepG2 cells and primary hepatocytes. Taken together, we speculate that a reduction in mitochondrial membrane potential and cytotoxicity during excessive intracellular RSS-induced stress may be linked to the expression level of a CysSSCys-dependent antiporter.

CSE is abundantly expressed in the liver and kidneys but minimally expressed in the heart and skeletal muscles [14,33]. It seems likely that heart and skeletal muscle may not favor reducing conditions mediated by RSS because they are adversely affected by reductive stress [34,35]. Indeed, the present study showed that heart and skeletal muscle tissues were sensitive to excess intracellular RSS induced by CSE and dietary CysSSCys supplementation.

In a previous study, we found that there are a numerous aliphatic hydrocarbons containing sulfane sulfur atoms in garlic extracted by hexane and that exposure of mice to this hexane extract is capable of increasing the plasma concentrations of endogenous RSS such as CysSSH [36]. Although we did not examine tissue concentrations of CysSSH in that study, it is likely that excess tissue CysSSH is excreted into the extracellular space via CysSSCys-dependent antiporters.

Our study investigated an adaptive response to excess RSS in which CysSSH is exported from the cell via CysSSCys-dependent antiporters such as a SLC7A11. However, we suggest that CysSSH and GSSH are exported out of the cell by different transporters (Fig. 4B). Therefore, the extracellular efflux mechanism of RSS other than CysSSH should be investigated in future studies.

Reductive stress, the opposite of oxidative stress, is defined as a condition characterized by excessive increase in reducing substances such as GSH and NADH [37]. Reductive stress is thought to be involved in a variety of diseases as well as oxidative stress [38]. Some studies have reported that reductive stress is involved in cardiomyopathy and muscular dystrophy via abnormal protein aggregation and mitochondrial impairment [34,38,39]. The p*K*a of CysSSH, an RSS, is 4.34, which is significantly lower than that of GSH (approximately 9) and highly reactive in a non-enzymatic manner under physiological conditions [2,40,41]. In other words, although RSS are beneficial as antioxidants against oxidative stress, they also pose a high risk of causing reductive stress. This study demonstrated a system that effluxes excess RSS out of the cell. This system probably contributes as a safeguard against reductive stress. Therefore, we believe that our findings open new directions of research in diseases related to reductive stress as well as sulfur biology.

## Authorship

M.A, T.U, H.A, E.W., and A.N. conducted the experiments. Y.K. wrote the original draft of the manuscript and M.A, and T.U. designed the experiments. Y.S. provided methodological support. N.A., T.A. and M.N. provided useful information and theoretical support. Y.K. supervised the study. All authors interpreted the data and assisted with editing the manuscript.

## Declaration of competing interest

The authors declare that they have no known competing financial interests or personal relationships that could have appeared to influence the work reported in this paper.

## Data Availability

The authors do not have permission to share data.
