# Supplementary material for: Cystine-dependent antiporters buffer against excess intracellular reactive sulfur species-induced stress
Source: Redox Biol. 2022 Oct 17;57:102514. doi: 10.1016/j.redox.2022.102514 (PMC9594640; doi:10.1016/j.redox.2022.102514)
Supplement: Multimedia component 1 [file mmc1.docx]

Supplementary data

Fig. S1. *Cse* mRNA expression levels.

Fig. S2. Effect of high expression of CSE proteins in the heart on the production of CysSH and their per/polysulfides.

Fig. S3. Intra- and extra-cellular concentrations of cysteine and glutathione in WT and CSE Tg mice.

Fig. S4. Effects of Na_2_S_4_ on intra- and extra-cellular concentrations of endogenous sulfur nucleophiles in primary hepatocytes.

Fig. S5. Effects of Na_2_S on intra- and extra-cellular concentrations of sulfur nucleophiles in primary hepatocytes.

Fig. S6. Effects of Na_2_S_4_ on intracellular and extracellular concentrations of endogenous persulfides in primary rat astrocytes.

Fig. S7. Effect of ABCC inhibitor MK571 against intracellular and extracellular sulfur nucleophile transitions.

Fig. S8. Effect of different extracellular amino acids on CysSSH export from Hepg2 cells.

Fig. S9. Effect of sulfasalazine treatment on CysSSH export from HepG2 and HEK 293 cells.

Fig. S10. Effects of CysSSSCys on intra- and extra-cellular concentrations of CysSSH in WT and SLC7A11 KO HepG2 cells.

Fig. S11. Effect of Na_2_S_4_ treatment on mitochondrial membrane potential in primary hepatocytes.

Fig. S12. Effect of high cystine diet on plasma sulfur nucleophile levels of WT and CSE Tg.

Fig. S13. Liver and quadricep muscle of WT and CSE Tg mice fed a regular diet.

Fig. S14. The plasma levels of alanine aminotransferase levels of WT and CSE Tg fed on a high cystine diet.

Fig. S15. Effect of high cystine diet on cardiac function of WT and CSE Tg.

Fig. 16. Effect of high cystine diet administration on survival of CSE Tg mice.

**Supplemental material and methods**

*1. Real-time polymerase chain reaction*

*2. Preparation and culture of rat astrocytes*

**Supplemental references**

Fig. S1. *Cse* mRNA expression levels.

*Cse* mRNA expression levels in the heart and skeletal muscle of wild-type (WT) or CSE Tg mice were analyzed using real-time-PCR. Data are shown as mean ± SEM (n = 3, each genotype). ***p* < 0.01.

Fig. S2. Effect of high expression of CSE proteins in the heart on the production of CysSH and their per/polysulfides. One mM of cystathionine (C) or CysSSCys (D) was incubated with protein from heart of WT mice and Tg mice overexpressing CSE in 100 mM Hepes buffer (pH 7.5) containing 100 μM pyridoxal phosphate at 37°C for 30 min, and then CysSH, CysSSH, and CysSSSH were measured by HPE-IAM labeling LC-MS/MS analysis. Data are shown as mean ± SEM (n = 3, each genotype). ***p* < 0.01.

Fig. S3. Intra- and extra-cellular concentrations of cysteine and glutathione in WT and CSE Tg mice. LC-ESI-MS/MS was used to quantitate percentage changes in (A) cysteine (CysSH), and (B) glutathione (GSH) concentrations in the low-molecular-weight fractions of liver, kidney, and heart lysates, and in plasma. Data represent the mean ± SEM (n = 3) and are expressed relative to the baseline measured in WT mice. *p < 0.05. Sulfur nucleophile concentrations (pmol/mg) in liver tissue from WT mice: CysSH (55.4 ± 12.7) and GSH (1845 ± 307); kidney tissue from WT mice: CysSH (2642 ± 63) and GSH (19.8 ± 1.7); heart tissue from WT mice: CysSH (24.3 ± 1.6) and GSH (641 ± 73). Sulfur nucleophile concentrations (nM) in plasma from WT mice: CysSH (7184 ± 2016) and GSH (2476 ± 396).

Fig. S4. Effects of Na_2_S_4_ on intra- and extra-cellular concentrations of endogenous sulfur nucleophiles in primary hepatocytes. (A and B) Cells were treated with serum-free WME containing 0 (control), 50, or 200 μM Na_2_S_4_ for 1 h, washed three times, and incubated in fresh serum-free WME. At the indicated times, (A) cells and (B) medium were collected and their persulfide concentrations quantified using LC-ESI-MS/MS. Data represent the mean ± SEM (*n* = 3). ***p* < 0.01, N.S.: not significant.

Fig. S5. Effects of Na_2_S on intra- and extra-cellular concentrations of sulfur nucleophiles in primary hepatocytes. (A and B) Cells were treated with serum-free WME containing 0 (control), 400, or 1000 μM Na_2_S for 1 h, washed three times, and incubated in fresh serum-free WME. At the indicated times, (A) cells and (B) medium were collected and their persulfide concentrations quantified using LC-ESI-MS/MS. Data represent the mean ± SEM (*n* = 3)

Fig. S6. Effects of Na_2_S_4_ on intracellular and extracellular concentrations of endogenous persulfides in primary rat astrocytes. (A and B) Cells were treated with serum-free DMEM containing 0 (control) or 100 μM Na_2_S_4_ for 1 h, washed three times, and incubated in fresh serum-free DMEM. At the indicated times, (A) cells and (B) medium were collected and their thiol and persulfide concentrations quantified using LC-ESI-MS/MS. Data represent the mean ± SEM (n = 3). **p < 0.01.

Fig. S7. Effect of ABCC inhibitor MK571 against intracellular and extracellular sulfur nucleophile transitions. (A and B) Primary mouse hepatocytes prepared from wild-type mice were incubated in serum-free WME alone (control) or serum-free WME containing 100 μM MK571. (A) After 12 h incubation, the cells were sonicated and incubated with 1 mM HPE-IAM at 37°C for 30 min to yield HPE-AM adducts for detecting intracellular sulfur nucleophiles. Each value is the mean ± SEM for the three independent experiments. ***p* < 0.01 (B) The WME media were collected at the indicated times and incubated with 5 mM HPE-IAM at 37°C for 30 min to yield HPE-AM adducts for extracellular sulfur nucleophiles detection. Each value is the mean ± SEM for the three independent experiments. **p* < 0.05, ***p* < 0.01.

Fig. S8. Effect of different extracellular amino acids on CysSSH export from Hepg2 cells. HepG2 cells were incubated with 100 µM Na_2_S_4_ for 1 h. After washing, the cells were incubated in indicated media for 3 h. The conditioned culture media were collected and reacted with HPE-IAM. Each value is the mean ± SEM for the three independent experiments. AA-1, MEM Essential Amino Acids Solution; AA-2, MEM Non-essential Amino Acids Solution; CysSSCys (100 µM), cystine; Arg (600 µM), arginine; His (200 µM), histidine; Iso (400 µM), Isoleucine; Leu (400 µM), leucine; Lys (400 µM), lysine; Met (100 µM), methionine; Phe (200 µM), phenylalanine; Thr (400 µM), threonine; Try (50 µM), tryptophan; Tyr (200 µM), tyrosine; Val (400 µM), valine.

Fig. S9. Effect of sulfasalazine treatment on CysSSH export from HepG2 and HEK 293 cells. (A) HepG2 cells and (B) HEK293 cells were treated with serum-free DMEM containing 100 μM Na_2_S_4_ for 1 h, followed by wash three times. Then, the cells were incubated with DMEM (control), HBSS, HBSS + CysSSCys, or HBSS + CysSSCys + sulfasalazine for 3 h. The conditioned culture media were collected and reacted with HPE-IAM. Each value is the mean ± SEM for the three independent experiments. ***p* < 0.01, compared with control.

Fig. S10. Effects of CysSSSCys on intra- and extra-cellular concentrations of CysSSH in WT and SLC7A11 KO HepG2 cells. (A and C) Intracellular and (B and D) extracellular concentrations of CysSSH from (A and B) WT and (C and D) SLC7A11-KO HepG2 cells treated with DMEM containing 0 (control), 500, or 1000 μM CysSSSCys for 1 h. After treatment, cells were washed three times and incubated with HBSS containing 100 µM CysSSCys, then cells and media were collected at the indicated time points and their CysSSH concentrations quantified using LC-ESI-MS/MS. Data represent the mean ± SE (*n* = 3).

Fig. S11. Effect of Na_2_S_4_ treatment on mitochondrial membrane potential in primary hepatocytes. (A and B) Primary hepatocytes were loaded with JC-1 and then treated with Na_2_S_4_ at the set concentration for 45 min. (A) JC-1 indicates green and red, while Hochest 33342 indicates blue. Scale bars: 50 μm. (B) The average ratio of JC-1 red-green fluorescence was quantified. Each value is the mean ± SEM for the three independent experiments. **p* < 0.05.

Fig. S12. Effect of high cystine diet on plasma sulfur nucleophile levels of WT and CSE Tg. (A-C) WT and Tg mice fed on a diet supplemented with 5% CysSSCys for 2 weeks. (A) CysSH, (B) GSH, and (C) GSSH levels in plasma (n = 3, each group).

Fig. S13. Liver and quadricep muscle of WT and CSE Tg mice fed a regular diet. Stereoscopic images and weights of (A) liver and (B) quadricep muscle. Data represent the mean ± SEM (*n* = 3, each genotype).

Fig. S14. The plasma levels of alanine aminotransferase levels of WT and CSE Tg fed on a high cystine diet. WT and Tg mice fed on a diet supplemented with 5% CysSSCys for 2 weeks. The plasma levels of alanine aminotransferase (ALT) levels (n = 5, each genotype).

Fig. S15. Effect of high cystine diet on cardiac function of WT and CSE Tg. WT and Tg mice fed on a diet supplemented with 5% CysSSCys for 2 weeks. (A) Ejection fraction (EF), (B) fractional shortening (FS), and (C) heart rate (HR) were measured by echocardiographic analysis. (n = 5, each genotype).

Fig. 16. Effect of high cystine diet administration on survival of CSE Tg mice. WT and Tg mice fed on a diet supplemented with 5% CysSSCys for 3 weeks. (A) Body weight change and (B) survival rate was measured. Data are shown as mean ± SEM (n = 3, each genotype).**p* < 0.05.

**Supplemental material and methods**

*1. Real-time polymerase chain reaction*

Total RNA from the heart and skeletal muscle of WT or CSE Tg mice using an RNeasy Lipid Tissue Mini Kit, and cDNA was synthesized using a High-Capacity cDNA Reverse Transcription Kit (Applied Biosystems, CA, USA), following the manufacturers’ protocols. Real-time PCR was performed using Power SYBR Green PCR Master Mix (Applied Biosystems) with a 7500 Real-Time PCR System (Applied Biosystems). The following PCR primers were used: forward 5′- CTTGCTGCCACCATTACG-3′ and reverse 5′- TTCAGATGCCACCCTCCT-3′ for *Cse*, and forward 5′-GGAGAATGGGAAGCCGAACA-3′ and reverse 5′-TCCTTGCTGAAGGACATATCTGACA -3′ for β2-microglobulin (*B2m*). The PCR conditions were as follows: 50°C for 2 min, 95°C for 10 min, and 45 cycles of 95°C for 15 s and 60°C for 1 min. Melting curve analysis was conducted to ensure amplification of a single product. The *Cse* and *B2m* mRNA levels in each RNA sample were determined using the relative standard curve method. Changes in *Cse* expression were assessed after the fluorescence intensity of its PCR product had been normalized relative to that of *B2m.*

*2. Preparation and culture of rat astrocytes*

Astrocytes were isolated from the cortex of Sprague Dawley Rats at embryonic day19 of gestation as described previously [1], seeded at a density of 8 × 10^4^ cells/cm^2^ on 6-well plates (AGC techno glass, Shizuoka, Japan) in Dulbecco's Modified Eagle Medium (DMEM) containing 10% fetal bovine serum, 2 mM L-alanyl-L-glutamine, 100 units/mL penicillin, and 100 μg/mL streptomycin, then cultured at 37°C in a humidified atmosphere with 5% CO_2_. Twenty-four hours after seeding, cells were used for subsequent assays with serum-free DMEM.

**Supplemental references**

[1] G. Zhao, M.P. Flavin, Differential sensitivity of rat hippocampal and cortical astrocytes to oxygen-glucose deprivation injury, Neurosci Lett 285(3) (2000) 177-80.
